# Enhanced Antioxidant and Antimicrobial Activities of an Ultrasound‐Optimized Green Tea–Rosemary Extract for Cosmetic Preservation

**DOI:** 10.1111/jocd.70972

**Published:** 2026-06-11

**Authors:** Anjan Kumar Singh, Abdul R. Mkia, Ali Fawzi Al‐Hussainy, Shaker Mohammed, Aashna Sinha, Subhashree Ray, Shayan Mahmoodi

**Affiliations:** ^1^ Sharda School of Bio‐Science & Technology Sharda University Greater Noida India; ^2^ Faculty of Allied Medical Sciences, Hourani Center for Applied Scientific Research Al‐Ahliyya Amman University Amman Jordan; ^3^ College of Pharmacy Ahl Al Bayt University Kerbala Iraq; ^4^ College of Pharmacy The Islamic University Najaf Iraq; ^5^ Department of Medical Analysis, Medical Laboratory Technique College The Islamic University of Al Diwaniyah Al Diwaniyah Iraq; ^6^ Division of Research and Innovation, School of Applied and Life Sciences Uttaranchal University Dehradun India; ^7^ Department of Biochemistry, IMS and SUM Hospital Siksha ‘O’ Anusandhan (Deemed to be University) Bhubaneswar India; ^8^ Department of Chemistry, Young Research Club Tehran University Tehran Iran

**Keywords:** antioxidant and antimicrobial activity, *Camellia sinensis*, cosmetic preservation, *Rosmarinus officinalis*, ultrasound‐assisted extraction

## Abstract

**Background:**

Growing concerns regarding the safety and environmental impact of synthetic preservatives have increased interest in natural, plant‐derived alternatives for cosmetic formulations. Polyphenol‐rich botanical extracts are particularly attractive due to their combined antioxidant and antimicrobial properties.

**Aims:**

This study aimed to develop and evaluate an ultrasound‐optimized polyphenol‐rich extract from a combination of *Camellia sinensis* (green tea) and *Rosmarinus officinalis* (rosemary) as a potential multifunctional preservative for cosmetic applications.

**Patients/Methods:**

A 1:1 blend of green tea and rosemary was subjected to ultrasound‐assisted extraction (UAE) to maximize phenolic recovery. The extract was characterized by measuring total phenolic content and analyzing key phytochemical markers using HPLC‐DAD. Antioxidant activity was assessed using β‐carotene bleaching inhibition, while antimicrobial activity was evaluated against *Staphylococcus aureus* , *Escherichia coli* , *Pseudomonas aeruginosa* , and *Candida albicans* . Minimum inhibitory concentrations and time‐kill kinetics were determined. The extract was further incorporated into a model cosmetic emulsion to evaluate its preservative performance during storage.

**Results:**

The optimized extract exhibited a high total phenolic content (152.8 ± 5.3 mg GAE/g) and higher yield compared with individual plant extracts. HPLC‐DAD confirmed the presence of catechin derivatives and carnosic acid, indicating a complementary phytochemical profile. Strong antioxidant activity was observed with β‐carotene inhibition of 78.4%. The extract demonstrated broad‐spectrum antimicrobial activity with minimum inhibitory concentrations ranging from 0.51 to 2.04 mg/mL. Time‐kill assays showed rapid microbial inactivation, and the cosmetic emulsion containing the extract maintained oxidative stability and microbial safety during storage.

**Conclusions:**

The ultrasound‐optimized green tea–rosemary extract demonstrated significant antioxidant and antimicrobial activities and effectively enhanced the stability of a cosmetic formulation. These findings suggest that this natural extract could serve as a promising eco‐friendly preservative for clean‐label cosmetic products.

## 1 Introduction

The cosmetic industry is increasingly pivoting towards natural, sustainable ingredients to meet consumer demand for clean‐label products that ensure microbial safety and oxidative stability without synthetic additives [1]. Preservatives are essential in cosmetic formulations to prevent microbial contamination and product degradation, which can cause skin irritation or spoilage [2]. Synthetic preservatives like parabens and formaldehyde releasers have raised concerns due to potential health risks, including endocrine disruption and allergenicity, driving the exploration of plant‐derived alternatives [3]. Polyphenol‐rich extracts from *Camellia sinensis* (green tea) and *Rosmarinus officinalis* (rosemary) are promising due to their antioxidant, antimicrobial, and anti‐inflammatory properties, positioning them as multifunctional candidates for cosmetic preservation [4]. These properties align with the growing trend of eco‐friendly cosmetics, addressing both consumer safety and environmental sustainability [5].

Polyphenols, secondary plant metabolites, exhibit potent antioxidant activity by neutralizing reactive oxygen species (ROS) through electron donation, protecting skin from oxidative stress and aging [6]. Green tea is rich in catechins, particularly epigallocatechin gallate (EGCG), which contributes to high total phenolic content (TPC) and robust radical‐scavenging capacity [7]. Rosemary, on the other hand, contains carnosic acid and rosmarinic acid, offering lipophilic antioxidant and antimicrobial effects against common cosmetic contaminants [8]. Combining these plants enhances bioactivity, as polar catechins from green tea synergize with non‐polar rosemary diterpenes, broadening the spectrum of antioxidant and antimicrobial action [9].

High‐performance liquid chromatography coupled with a diode‐array detector (HPLC‐DAD) is widely applied for the characterization of plant polyphenols due to its high sensitivity and selectivity. The technique enables simultaneous separation and detection of multiple phenolic compounds while providing UV–visible spectral information useful for compound identification. In studies of botanical extracts, HPLC‐DAD is commonly employed to verify the presence of characteristic phenolic markers and to support the interpretation of biological activities associated with complex phytochemical mixtures. To verify the presence of representative phenolic constituents from both plant sources in the combined extract, chromatographic characterization was performed in the present study. Extraction efficiency plays a critical role in maximizing polyphenol yield and preserving bioactivity. Conventional techniques such as maceration often require long extraction times and large volumes of solvent, which reduces efficiency and sustainability. In contrast, green extraction technologies such as ultrasound‐assisted extraction (UAE) have emerged as effective alternatives [10].

Extraction efficiency is critical for maximizing polyphenol yield and bioactivity. Conventional methods like maceration are often inefficient and solvent‐intensive, whereas green technologies such as ultrasound‐assisted extraction (UAE) improve sustainability and efficacy [11]. UAE employs acoustic cavitation to disrupt plant cell walls, facilitating rapid release of bioactives with minimal thermal degradation [12]. Optimized UAE parameters (e.g., ethanol concentration, temperature, sonication time) have increased polyphenol yields from green tea to 20%–25%, with IC_50_ values as low as 0.4 mg/mL in antioxidant assays [13]. For rosemary, UAE enhances carnosic acid extraction, achieving TPC values competitive with synthetic antioxidants [14]. Combined green tea‐rosemary extracts benefit from UAE's ability to co‐extract polar and lipophilic compounds, yielding higher TPC and bioactivity than microwave or solvent‐based methods [15, 16].

Antimicrobial efficacy is vital for cosmetic preservatives, targeting pathogens like *Staphylococcus aureus* , *Escherichia coli* , *Pseudomonas aeruginosa* , and *Candida albicans* [17]. Green tea catechins disrupt microbial membranes via hydrogen bonding, while rosemary's phenolic acids inhibit efflux pumps and metabolic enzymes [18]. Blended extracts demonstrate lower minimum inhibitory concentrations (MICs, 0.5–2 mg/mL) due to enhanced mechanisms, outperforming individual extracts in broth microdilution assays [19]. These properties align with ISO 11930 standards, which mandate ≥ 2–3 log reductions in microbial counts over 28 days [20]. Recent formulations with 1%–2% blended extracts have met these criteria, ensuring microbial safety in emulsions under accelerated storage [21]. Antioxidant activity further enhances preservation by inhibiting lipid peroxidation, as evidenced by 70%–80% inhibition in β‐carotene bleaching assays [22]. Polyphenols also offer skin health benefits, mitigating UV‐induced ROS and inflammation via NF‐κB pathway suppression [23, 24].

Despite their potential, challenges in polyphenol stability and scalability persist. Sensitivity to pH, light, and oxidation necessitates advanced delivery systems like encapsulation [25]. Regulatory frameworks, such as EU Cosmetics Regulation 1223/2009, require rigorous safety assessments, with recent studies confirming the non‐irritating nature of green tea and rosemary extracts at cosmetic concentrations [26]. The clean beauty movement underscores the urgency of replacing synthetic preservatives, which contribute to environmental persistence, with sustainable alternatives [27]. UAE's reduced solvent and energy use supports this shift, aligning with green chemistry principles [28]. Prior studies on combined extracts report enhanced TPC and bioactivity but lack cosmetic‐specific validation, highlighting a research gap this study addresses [29]. By optimizing UAE for a 1:1 green tea‐rosemary blend and validating its efficacy per ISO 11930, this work advances sustainable cosmetic preservation, offering a multifunctional, eco‐friendly solution [30].

In this context, the present study aimed to optimize the extraction of a 1:1 blend of *Camellia sinensis* and *Rosmarinus officinalis* using ultrasound‐assisted extraction and to characterize its phenolic composition through chromatographic analysis. The antioxidant capacity and antimicrobial efficacy of the resulting extract were evaluated using multiple in vitro assays, and its preservative performance was further validated in a model cosmetic emulsion according to ISO 11930 guidelines. By integrating optimized extraction, phytochemical characterization, and functional validation, this work explores the potential of a green tea–rosemary extract as a multifunctional natural preservative for sustainable cosmetic formulations.

## 2 Materials and Methods

This section details the materials, experimental procedures, and analytical methods used to prepare, characterize, and evaluate a polyphenol‐rich extract obtained from a 1:1 (w/w) combination of *Camellia sinensis* (green tea) and *Rosmarinus officinalis* (rosemary) leaves. An optimized UAE protocol was employed, and the combined extract was compared with individual *C. sinensis* and *R. officinalis* extracts. All experiments were performed in triplicate, and methodologies adhered to ISO 11930 requirements for preservative efficacy testing in cosmetic formulations.

### 2.1 Materials

#### 2.1.1 Plant Material

Dried leaves of *Camellia sinensis* and *Rosmarinus officinalis* were obtained from a certified organic supplier. Leaves were air‐dried at 25°C for 7 days, ground to a fine powder using a laboratory grinder, and stored in airtight containers at 4°C until use.

#### 2.1.2 Chemicals and Reagents

Analytical‐grade ethanol (96%), potassium permanganate (KMnO_4_), gallic acid (≥ 98%), sodium carbonate, 2,6‐dichlorophenolindophenol (DCPIP), β‐carotene, linoleic acid, Tween 40, and Folin–Ciocalteu reagent were purchased from Merck (Germany). Thin‐layer chromatography (TLC) plates (silica gel 60 F_254_) and mobile‐phase solvents (chloroform, methanol, acetic acid) were obtained from Sigma‐Aldrich (USA). Mueller–Hinton broth (MHB), Mueller–Hinton agar (MHA), and Sabouraud dextrose broth (SDB) were sourced from HiMedia (India). All chemicals were of analytical grade.

#### 2.1.3 Microorganisms

The antimicrobial activity assays employed *Staphylococcus aureus* (ATCC 25923), *Escherichia coli* (ATCC 25922), *Pseudomonas aeruginosa* (ATCC 27853), and *Candida albicans* (ATCC 10231), selected for their relevance as common cosmetic contaminants. Bacterial strains were maintained on MHA, and yeast cultures on SDB, at 4°C and subcultured prior to experiments.

#### 2.1.4 Standards and Controls

Gallic acid was used as the reference standard for TPC determination. Chloramphenicol (for bacterial assays) and fluconazole (for *C. albicans* ) served as positive antimicrobial controls. Sterile distilled water and 10% ethanol were used as negative controls. Individual *C. sinensis* and *R. officinalis* extracts were prepared under identical conditions to serve as comparators. A preservative‐free model cosmetic emulsion was prepared for the ISO 11930 challenge test, with extract concentrations selected based on preliminary solubility and compatibility screening (Table 1).

**TABLE 1:** Composition of the model cosmetic emulsion used in ISO 11930 preservative efficacy testing.

Component	Percentage (w/w)	Source
Water	70	Distilled
Glycerin	5	Merck, Germany
Cetyl alcohol	3	Sigma‐Aldrich, USA
Stearic acid	2	Sigma‐Aldrich, USA
Polysorbate 80	1.5	Merck, Germany
Polyphenol extract^a^	0.50–2.00	Prepared in this study
Xanthan gum	0.3	Sigma‐Aldrich, USA
Preservative‐free base	q.s. to 100	—

^a^ Concentration range used depending on the specific experimental design. The emulsion pH was adjusted to 5.5 using citric acid to mimic typical cosmetic formulations.

### 2.2 Extraction of Plant Polyphenols

Polyphenol‐rich extracts were prepared from *C. sinensis* , *R. officinalis* , and their 1:1 (w/w) combination using ultrasound‐assisted extraction. Extraction parameters were optimized via response surface methodology (RSM) with ethanol concentration, sonication time, and extraction temperature as independent variables. For each extraction, 100 g of powdered plant material was suspended in aqueous ethanol at the optimized concentration (v/v) and processed using an ultrasonic processor under the optimized temperature and time conditions. The resulting mixtures were filtered through Whatman No. 1 filter paper, concentrated under reduced pressure using a rotary evaporator at 45°C, and subsequently lyophilized to obtain dry extracts. Dried extracts were stored at −20°C in airtight containers until further analysis.

#### 2.2.1 Optimization and Justification of Extract Ratio

Preliminary experiments were conducted to determine the optimal blending ratio of *Camellia sinensis* and *Rosmarinus officinalis* for maximizing TPC and bioactivity. Ratios of 1:0, 3:1, 1:1, 1:3, and 0:1 (w/w) were prepared under identical UAE conditions and analyzed for TPC, DCPIP antioxidant activity, and minimum inhibitory concentration (MIC) against *Staphylococcus aureus* . The results (Table 2) indicated that the 1:1 ratio achieved the highest TPC and exhibited superior antioxidant and antimicrobial performance compared to other ratios, suggesting an enhanced effect between catechin‐rich *C. sinensis* and carnosic acid–rich *R. officinalis* . This ratio was therefore selected for subsequent experiments to balance polar and lipophilic phenolic compounds while maintaining industrial scalability.

**TABLE 2:** Preliminary screening of different *C. sinensis* and *R. officinalis* ratios.

Ratio (w/w)	TPC (mg GAE/g)	DCPIP IC_50_ (mg/mL)	MIC ( *S. aureus* , mg/mL)
1:00	132.56 ± 4.89	0.62 ± 0.04	0.78 ± 0.02
3:01	141.47 ± 5.12	0.51 ± 0.03	0.65 ± 0.02
1:01	152.83 ± 5.27	0.42 ± 0.03	0.51 ± 0.01
1:03	146.92 ± 4.98	0.49 ± 0.03	0.56 ± 0.02
0:01	118.42 ± 4.12	0.78 ± 0.05	0.92 ± 0.03

### 2.3 Phytochemical Profiling

Thin‐layer chromatography (TLC) was used to perform qualitative phytochemical profiling of the extracts. Analyses were conducted on silica gel 60 F_254_ plates (Merck) using a mobile phase composed of chloroform:methanol:acetic acid (85:15:2, v/v/v). Aliquots (10 μL) of each extract solution (10 mg/mL in methanol) were applied as spots, and chromatograms were developed to a solvent front of 8 cm. Plates were examined under ultraviolet light at 254 nm and 366 nm, and subsequently sprayed with 1% ferric chloride (FeCl_3_) reagent to visualize phenolic compounds. Retention factor (*R*_*f*_) values were calculated according to the standard formula [31]:

(1) $$R_{f}=\frac{\text{Distance travelled}\;\mathrm{by}\;\text{compound}\;\left(\mathrm{cm}\right)}{\text{Distance travelled}\;\mathrm{by}\;\text{solvent front}\;\left(\mathrm{cm}\right)}$$

### 2.4 HPLC Characterization and Quantification of Major Phenolic Compounds

High‐performance liquid chromatography (HPLC) analysis was performed to achieve a more accurate characterization and quantification of the principal phenolic constituents present in the optimized extract obtained from the 1:1 (w/w) mixture of *Camellia sinensis* and *Rosmarinus officinalis* .

Chromatographic analyses were carried out using an HPLC system equipped with a quaternary pump, autosampler, column oven, and diode‐array detector (DAD). Separation of phenolic compounds was achieved on a reverse‐phase C18 analytical column (250 × 4.6 mm, 5 μm particle size) maintained at 30°C.

The mobile phase consisted of:

- **Solvent A:** water containing 0.1% formic acid
- **Solvent B:** acetonitrile containing 0.1% formic acid

A gradient elution program was applied as follows:

- 0–5 min: 10% B
- 5–15 min: 10%–25% B
- 15–25 min: 25%–40% B
- 25–30 min: 40%–60% B

The flow rate was maintained at 1.0 mL/min, and the injection volume was 20 μL. Detection of phenolic compounds was performed using a diode‐array detector at 280 nm, a wavelength commonly used for monitoring catechins and related phenolics.

Prior to analysis, extract samples were dissolved in methanol (10 mg/mL), filtered through 0.22 μm PTFE syringe filters, and injected into the HPLC system.

Identification of compounds was carried out by comparing retention times and UV absorption spectra with those of authentic reference standards, including:

- catechin
- epigallocatechin gallate (EGCG)
- rosmarinic acid
- carnosic acid

Quantification was performed using external calibration curves constructed from standard solutions at five concentration levels (5–100 μg/mL). Calibration curves showed excellent linearity (*R* ^2^ > 0.998). Results were expressed as mg of compound per g of dry extract (mg/g DE). All analyses were conducted in triplicate, and results are reported as mean ± standard deviation. HPLC analysis in this study was primarily conducted to confirm the presence and quantify representative phenolic markers originating from both plant sources. Therefore, quantitative results are summarized in tabular form rather than presenting full chromatographic fingerprints.

### 2.5 Total Phenolic Content

The TPC of each extract was determined by potassium permanganate (KMnO_4_) titration. In brief, 0.5 g of extract was dissolved in 50 mL of distilled water. A 10 mL aliquot of this solution was titrated against 0.01 N KMnO_4_ until a persistent pale pink endpoint was observed. TPC was expressed as milligrams of gallic acid equivalents per gram of dry extract (mg GAE/g), using a gallic acid calibration curve (0–100 μg/mL). All measurements were performed in triplicate.

### 2.6 Antioxidant Activity

Antioxidant capacity was evaluated using three complementary methods: DCPIP reduction assay, β‐carotene bleaching assay, and oxidative stability testing. All assays were conducted in triplicate.

#### 2.6.1 DCPIP Reduction Assay

The DCPIP (2,6‐dichlorophenolindophenol) assay was used to measure the electron‐donating ability of the extracts. Extract solutions (0.1–1.0 mg/mL) were prepared in phosphate buffer (pH 7.0) and mixed with 0.1 mM DCPIP solution. Reaction mixtures were incubated at 25°C for 30 min, and absorbance was recorded at 517 nm using a UV–Vis spectrophotometer. Ascorbic acid was used as the reference antioxidant. The percentage reduction of DCPIP was calculated as [32]:

(2) $$\;\text{Reduction}\%=\frac{A_{0}-A_{s}}{A_{0}}\times 100$$

where *A*_0_ is the absorbance of the control (without extract) and *A*_s_ is the absorbance in the presence of the extract.

#### 2.6.2 β‐Carotene Bleaching Assay

The β‐carotene bleaching method was used to assess the inhibition of lipid peroxidation. A β‐carotene–linoleic acid emulsion was prepared by dissolving β‐carotene in chloroform, adding linoleic acid and Tween 40, and evaporating the chloroform under nitrogen. The residue was emulsified with distilled water to obtain a stable suspension. Extract solutions (0.1–1.0 mg/mL) were mixed with the emulsion and incubated at 50°C. Absorbance at 470 nm was measured at 20‐min intervals over a 120‐min period. Butylated hydroxytoluene (BHT) served as the positive control. Antioxidant activity was calculated using [33]:

(3) $$\;\text{Inhibition}\%=\frac{A_{t}}{A_{0}}\times 100$$

where *A*_t_ is the absorbance at a given time interval and *A*_0_ is the initial absorbance.

#### 2.6.3 Oxidative Stability Test

The oxidative stability of a model oil‐in‐water cosmetic emulsion containing the plant extracts was evaluated under accelerated storage conditions. Samples were stored at 40°C for 28 days, and the peroxide value (PV) was determined at defined intervals to monitor the progression of lipid oxidation. This assay provided an estimate of the antioxidant effectiveness of the polyphenol‐rich extract within the emulsion system.

In parallel with PV determination, the physical stability of the formulations was systematically assessed during storage. Visual appearance, color, odor, and pH were monitored, and the presence of any phase separation was evaluated both visually and by centrifugation (3000 rpm, 10 min). No visible phase separation, creaming, or significant pH variation was observed throughout the test period, indicating that incorporation of the extract did not adversely affect the physical integrity of the cosmetic emulsion under accelerated aging conditions.

### 2.7 Antimicrobial Activity

The antimicrobial activity of the extracts was evaluated by determining MIC and minimum bactericidal/fungicidal concentration (MBC/MFC) using the broth microdilution method. The protocol followed the guidelines of the Clinical and Laboratory Standards Institute (CLSI) M07‐A10 for bacteria and M27‐A3 for yeasts.

Serial two‐fold dilutions of each extract (0.125–8.0 mg/mL) were prepared in MHB for bacteria or SDB for yeast in sterile 96‐well microplates. Each well was inoculated with microbial suspensions adjusted to approximately 10^5^ CFU/mL. Plates were incubated at 37°C for bacteria or 28°C for yeast under static conditions for 24–48 h. MIC was defined as the lowest extract concentration that completely inhibited visible growth. For MBC/MFC determination, 10 μL aliquots from wells showing no visible growth were plated onto Mueller–Hinton agar (MHA) for bacteria or Sabouraud dextrose agar for yeast. Plates were incubated under the same conditions, and the lowest concentration yielding no colony growth was recorded as the MBC or MFC (Table 3).

**TABLE 3:** Experimental setup for MIC and MBC/MFC determination.

Parameter	Description
Method	Broth microdilution (CLSI M07‐A10 for bacteria; CLSI M27‐A3 for yeast)
Microbial inoculum	~10^5^ CFU/mL in appropriate broth medium
Test concentrations	0.125–8.0 mg/mL (two‐fold serial dilutions)
Media	MHB for bacteria; SDB for yeast
Incubation conditions	37°C (bacteria); 28°C (yeast)
MIC determination	Lowest concentration with no visible growth
MBC/MFC determination	Plating 10 μL from non‐turbid wells onto agar; lowest concentration yielding no growth

#### 2.7.1 Cellular Leakage Assay

Cellular leakage was assessed by quantifying the release of intracellular components absorbing at 260 nm (nucleic acids) and 280 nm (proteins). Microbial suspensions were exposed to each extract at twice the MIC for a fixed exposure period under shaking conditions. After centrifugation, supernatants were analyzed spectrophotometrically. Untreated controls and solvent controls were included for baseline reference.

#### 2.7.2 Electrical Conductivity Assay

Electrical conductivity measurements were performed to evaluate membrane integrity disruption. Microbial suspensions were treated with extracts at twice the MIC, and conductivity readings were recorded at 0, 2, and 4 h using a calibrated conductivity meter (Hanna HI‐98192). Untreated cultures were analyzed in parallel to correct for background conductivity.

### 2.8 Time‐Kill Kinetics

Time‐kill assays were performed against *S. aureus* and *C. albicans* . Microbial suspensions (10^6^ CFU/mL) were incubated with extracts at MIC, 2× MIC, and 4× MIC in appropriate broth media. At designated time points, aliquots were removed, serially diluted, and plated on selective agar media to enumerate survivors (Table 4).

**TABLE 4:** Sampling schedule for time‐kill kinetics assays.

Time point (h)	Procedure
0	Inoculation of extract‐treated cultures at MIC, 2× MIC, and 4× MIC
2	Sampling, serial dilution, and plating on appropriate agar
4	Sampling, serial dilution, and plating
8	Sampling, serial dilution, and plating
12	Sampling, serial dilution, and plating
24	Sampling, serial dilution, and plating

### 2.9 Preservative Efficacy Test (ISO 11930)

Preservative efficacy was evaluated according to ISO 11930 using the model cosmetic emulsion described in Section 2.1.4. Formulations containing selected concentrations of the plant extract were challenged with representative microorganisms specified in ISO 11930, including Gram‐positive and Gram‐negative bacteria, yeast, and mold. The initial microbial load was adjusted to approximately 10^5^–10^6^ CFU/g for each test organism. Inoculated samples were stored at 22.5°C ± 2.5°C and analyzed on days 0, 7, 14, 21, and 28.

Microbial enumeration was performed using standard plate count methods, and log_10_ reductions relative to the initial inoculum were calculated. Preservative performance was assessed based on the acceptance criteria defined in ISO 11930, taking into account both the magnitude of microbial reduction at intermediate time points and the absence of significant regrowth by the end of the test period. This standardized challenge test enabled an objective evaluation of the preservative potential of the extract‐containing formulation in a cosmetic matrix.

### 2.10 Statistical Analysis

All experiments were performed in triplicate, and data were expressed as mean ± standard deviation (SD). Statistical analyses were conducted using SPSS software version 26. One‐way analysis of variance (ANOVA) followed by Tukey's post hoc test was applied to assess significant differences among means (*p* < 0.05). Pearson correlation analysis was used to evaluate relationships between total phenolic content, antioxidant capacity, and antimicrobial activity.

## 3 Results and Discussion

This section presents the findings from the evaluation of a polyphenol‐rich extract derived from a 1:1 (w/w) combination of *Camellia sinensis* (green tea) and *Rosmarinus officinalis* (rosemary) leaves, prepared using an optimized UAE method. The results cover extraction yield, phytochemical profiling via thin‐layer chromatography (TLC), TPC, antioxidant activity, antimicrobial efficacy, Time‐kill kinetics, preservative efficacy per ISO 11930 standards, mechanistic assays (cellular leakage and electrical conductivity), and oxidative stability to link antioxidant activity to cosmetic preservation. The combined extract was compared against individual *C. sinensis* and *R. officinalis* extracts to demonstrate enhanced effects. Pearson correlation analysis was conducted to explore relationships between TPC, antioxidant activity, and antimicrobial efficacy. All experiments were performed in triplicate, and data are reported as mean ± standard deviation (SD). Statistical significance was assessed using one‐way ANOVA with Tukey's post hoc test (*p* < 0.05).

### 3.1 Extraction Yield and TLC Profiling

The UAE of the combined *C. sinensis* and *R. officinalis* extract yielded a mean extraction efficiency of 21.47% ± 1.32% (w/w), higher than individual extracts: 18.62% ± 1.14% for *C. sinensis* and 16.45% ± 0.98% for *R. officinalis* . Compared to literature values for green tea (10%–15%, [34]) and rosemary (12%–18%, [35]), the combined extract's higher yield reflects UAE's cavitation effects, which enhance cell wall disruption and polyphenol solubilization. The optimized conditions (65% ethanol, 45°C, 20 min) likely facilitated the extraction of both polar catechins and lipophilic carnosic acid, contributing to the elevated yield. The low SD indicates robust reproducibility, critical for industrial applications.

TLC profiling revealed four distinct spots for the combined extract (Table 5), confirming a diverse polyphenolic profile. Spot 1 (*R*_*f*_ = 0.32) showed blue fluorescence under UV (366 nm) and dark blue with FeCl_3_, consistent with catechin. Spot 2 (*R*_*f*_ = 0.45) exhibited greenish fluorescence and brown coloration, indicative of epigallocatechin gallate (EGCG). Spot 3 (*R*_*f*_ = 0.58) displayed faint blue fluorescence and dark brown, suggesting gallic acid. Spot 4 (*R*_*f*_ = 0.67), unique to rosemary, showed yellowish fluorescence and dark green, corresponding to carnosic acid. Individual *C. sinensis* extracts lacked carnosic acid, while *R. officinalis* extracts lacked catechins, confirming the combined extract's enhanced chemical diversity. These *R*_*f*_ values align with prior studies (e.g., Amarowicz et al. [36]), validating the method's reliability.

**TABLE 5:** TLC phytochemical profiling results (combined extract).

Spot no.	*R*_*f*_ value	Color (UV 366 nm)	Color (FeCl_3_ spray)	Tentative compound
1	0.32	Blue fluorescence	Dark blue	Catechin
2	0.45	Greenish	Brown	Epigallocatechin gallate
3	0.58	Faint blue	Dark brown	Gallic acid
4	0.67	Yellowish	Dark green	Carnosic acid

*Note:* The combined extract's diverse profile suggests potential enhanced interactions, enhancing its bioactivity compared to individual extracts.

The enhanced extraction yield and diverse TLC profile underscore the methodological advantages of UAE in producing a polyphenol‐rich extract with potential industrial scalability. The higher yield of the combined extract compared to individual extracts highlights the efficacy of blending *C. sinensis* and *R. officinalis* , due to complementary cell wall structures that facilitate greater release of bioactive compounds during sonication. The TLC results confirm the presence of structurally diverse polyphenols, which are critical for downstream bioactivity, as their varied polarities and functional groups enable multiple modes of action. The reproducibility of *R*_*f*_ values across replicates (SD < 0.02) validates the robustness of the TLC method, while the identification of carnosic acid alongside catechins positions this extract as a novel candidate for cosmetic applications, offering a broader spectrum of bioactive molecules compared to single‐plant extracts reported in prior studies.

### 3.2 HPLC Identification and Quantification of Major Bioactive Phenolics

High‐performance liquid chromatography coupled with diode‐array detection (HPLC‐DAD) was employed to obtain a more reliable characterization of the principal phenolic constituents present in the optimized 1:1 extract of *Camellia sinensis* and *Rosmarinus officinalis* . In contrast to the preliminary qualitative insights obtained through TLC screening, HPLC provided a higher level of analytical resolution and enabled the targeted identification and quantification of representative phenolic markers derived from both botanical sources. Under the optimized chromatographic conditions, the phenolic compounds were well separated on a reverse‐phase C18 column, producing reproducible peaks with clear baseline resolution. Replicate injections demonstrated high analytical consistency, with minimal variation in retention time and peak area across runs, indicating good method stability and repeatability.

Peak identification was carried out through a systematic comparison of chromatographic retention times and diode‐array UV absorption spectra with those obtained from authentic analytical standards analyzed under identical experimental conditions. Catechin and epigallocatechin gallate (EGCG) were identified as the dominant flavan‐3‐ol derivatives characteristic of *C. sinensis* , whereas rosmarinic acid and carnosic acid were detected as the principal phenolic compounds associated with *R. officinalis* . The UV spectral profiles recorded by the diode‐array detector further supported compound identification, with catechin derivatives displaying characteristic absorption maxima around 278–280 nm and rosmarinic acid exhibiting a typical phenolic absorption pattern consistent with literature reports. The simultaneous detection of these compounds within a single chromatographic run confirmed that the optimized extraction process effectively recovered phenolic constituents from both plant matrices.

Quantitative determination of the identified compounds was performed using external calibration with five‐point standard curves prepared from certified reference materials. Each calibration curve demonstrated excellent linearity over the tested concentration range, with correlation coefficients exceeding 0.998. The concentrations measured in the extract revealed that EGCG represented the most abundant catechin derivative contributed by *C. sinensis* , whereas carnosic acid was the predominant diterpenoid phenolic associated with rosemary. Catechin and rosmarinic acid were also detected at appreciable levels, indicating that the extract possesses a chemically diverse phenolic profile. The simultaneous presence of flavonoid‐type polyphenols and rosemary diterpenes reflects the complementary phytochemical contributions of the two plant species and supports the rationale behind the combined extraction strategy.

From a functional perspective, the coexistence of these phenolic constituents is particularly relevant to the biological activities investigated in this study. Catechins and EGCG are widely recognized for their strong antioxidant and antimicrobial activities, while rosmarinic acid and carnosic acid are known to contribute both radical‐scavenging capacity and antimicrobial effects in plant‐derived extracts. The HPLC results therefore provide analytical evidence that the optimized extract contains multiple bioactive phenolic markers previously associated with preservative and antioxidant activity. This chemically confirmed phenolic composition strengthens the mechanistic interpretation of the antimicrobial and antioxidant results reported in subsequent sections and supports the potential application of the extract as a multifunctional natural preservative in cosmetic formulations.

Table 6 summarizes the quantitative HPLC‐DAD analysis of the principal phenolic compounds detected in the optimized extract. The table reports the chromatographic retention time for each compound, the corresponding calibration equation obtained from external standards, the coefficient of determination (*R* ^2^) indicating calibration linearity, and the measured concentration expressed as mg per g of dry extract. The reported values represent the mean ± standard deviation of triplicate analyses. The consistency of retention times together with the high linearity of calibration curves confirms the robustness and reliability of the applied HPLC method for phenolic quantification.

**TABLE 6:** HPLC‐DAD quantification of major phenolic compounds in the optimized 1:1 extract of *Camellia sinensis* and *Rosmarinus officinalis* .

Compound	Retention time (min)	Calibration equation	*R* ^2^	Concentration (mg/g dry extract)
Catechin	8.41	*y* = 12.54*x* + 3.21	0.9991	17.8 ± 0.8
EGCG	12.73	*y* = 15.02*x* + 2.87	0.9989	36.4 ± 1.5
Rosmarinic acid	10.96	*y* = 11.36*x* + 1.94	0.9993	14.1 ± 0.6
Carnosic acid	18.28	*y* = 13.11*x* + 2.65	0.9987	22.5 ± 1.1

*Note:* Values represent mean ± standard deviation (*n* = 3).

### 3.3 Total Phenolic Content

TPC, measured via KMnO_4_ titration, was 152.83 ± 5.27 mg GAE/g for the combined extract, compared to 132.56 ± 4.89 mg GAE/g ( *C. sinensis* ) and 118.42 ± 4.12 mg GAE/g ( *R. officinalis* ). These values exceed typical green tea (100–150 mg GAE/g, [34]) and rosemary (80–120 mg GAE/g, [35]) extracts, likely due to UAE's efficiency and the enhanced phenolic contribution of both plants. The gallic acid standard curve (*R* ^2^ = 0.998, (*y* = 0.012*x* + 0.015)) ensured accurate quantification. The higher TPC in the combined extract suggests a greater pool of hydroxyl groups, which drive antioxidant and antimicrobial activities by donating electrons and disrupting microbial membranes [37].

The elevated TPC of the combined extract underscores the methodological advantages of UAE and the strategic selection of a dual‐plant matrix, enhancing the extract's potential for cosmetic applications. The significantly higher TPC compared to individual extracts (*p* < 0.05, ANOVA) reflects the complementary phenolic profiles of *C. sinensis* (rich in catechins) and *R. officinalis* (rich in carnosic acid), which together maximize phenolic yield without compromising compound stability. This is critical for industrial scalability, as higher TPC correlates with enhanced bioactivity, potentially reducing the required preservative concentration in formulations. The low SD values (5.27–4.12 mg GAE/g) across all extracts confirm the precision of the KMnO_4_ titration method, validated by the near‐perfect linearity of the standard curve. Compared to prior studies, the combined extract's TPC positions it as a competitive alternative to synthetic preservatives, offering a sustainable, bioactive ingredient. The results lay a foundation for subsequent analyses, demonstrating that the extract's phenolic richness is a key driver of its multifunctional properties in cosmetic preservation.

### 3.4 Antioxidant Activity

Antioxidant activity was assessed using DCPIP reduction, β‐carotene bleaching, and oxidative stability tests, hypothesizing that antioxidant activity reduces lipid peroxidation in cosmetic emulsions [38].

#### 3.4.1 DCPIP Reduction Assay

The combined extract exhibited an IC_50_ of 0.42 ± 0.03 mg/mL, significantly lower than 0.62 ± 0.04 mg/mL ( *C. sinensis* ), 0.78 ± 0.05 mg/mL ( *R. officinalis* ), and 0.52 ± 0.02 mg/mL (ascorbic acid) (*p* < 0.05). At 1.0 mg/mL, the combined extract achieved 92.35% ± 2.87% reduction, compared to 85.67% ± 2.14% (ascorbic acid), 80.23% ± 2.56% ( *C. sinensis* ), and 76.89% ± 2.33% ( *R. officinalis* ).

The superior activity can be attributed to the complementary contribution of phenolic constituents identified in the extract, particularly catechin derivatives and carnosic acid as confirmed by the HPLC analysis. The multiple hydroxyl groups of catechins enhance electron‐donating capacity, while the lipophilic diterpenoid structure of carnosic acid contributes to radical stabilization in lipid environments.

#### 3.4.2 β‐Carotene Bleaching Assay

The combined extract inhibited bleaching by 78.43% ± 3.22% at 1.0 mg/mL, compared to 88.92% ± 2.65% (BHT), 72.15% ± 3.01% ( *C. sinensis* ), and 68.47% ± 2.89% ( *R. officinalis* ) (*p* < 0.05). The activity reflects efficient scavenging of peroxyl radicals within the emulsion system. As indicated by the HPLC phytochemical characterization, the extract contains both catechin derivatives and rosemary phenolics such as carnosic acid, whose lipophilic nature enhances antioxidant protection in lipid phases [35].

#### 3.4.3 Oxidative Stability Test

The model emulsion with 2.0% combined extract had a peroxide value (PV) of 4.82 ± 0.37 meq/kg after 28 days at 40°C, compared to 6.45 ± 0.51 meq/kg ( *C. sinensis* ), 7.12 ± 0.59 meq/kg ( *R. officinalis* ), and 12.67 ± 0.89 meq/kg (control) (*p* < 0.01). This indicates superior inhibition of lipid oxidation, supporting the hypothesis that antioxidant activity enhances formulation stability. In addition to improved oxidative resistance, the extract‐containing formulations demonstrated acceptable physical stability throughout the test period. No visible phase separation or creaming was observed, including after centrifugation, and the pH remained within the acceptable cosmetic range. These results indicate that the polyphenol‐rich extract can be incorporated into cosmetic formulations without compromising physical stability, while simultaneously contributing to improved oxidative protection.

In addition to their preservative and oxidative stabilizing effects, the combined extracts of *Camellia sinensis* and *Rosmarinus officinalis* may also offer additional cosmetic benefits related to skin anti‐aging. Both plants are rich in polyphenolic compounds known for their antioxidant properties, which may help reduce oxidative stress associated with skin aging. Therefore, beyond improving formulation stability, this polyphenol‐rich combination may represent a multifunctional ingredient with potential value in anti‐aging cosmetic applications. However, dedicated biological studies would be required to confirm such effects in skin‐related models.

The antioxidant activity results highlight the combined extract's superior performance across multiple assays, positioning it as a promising candidate for cosmetic preservation. The DCPIP and β‐carotene assays demonstrate the extract's versatility in neutralizing both aqueous and lipid‐phase radicals, a critical attribute for stabilizing complex emulsions. The oxidative stability test's low PV underscores the extract's ability to maintain formulation integrity under accelerated aging conditions, outperforming individual extracts (*p* < 0.01). This synergy is due to the complementary chemical profiles of *C. sinensis* and *R. officinalis* , where polar catechins and lipophilic carnosic acid address diverse oxidative challenges. Compared to prior studies (e.g., Lobo et al. [38], reporting 70%–85% inhibition for green tea), the combined extract's performance is competitive, with the added benefit of rosemary's lipophilic antioxidants. The low SD values (e.g., 2.87%–3.22%) across assays confirm methodological precision, while the statistically significant improvements over individual extracts validate the rationale for combining these plants. These findings suggest that the extract's antioxidant properties could reduce reliance on synthetic stabilizers, offering a sustainable alternative for cosmetic formulations.

### 3.5 Antimicrobial Activity

MIC and MBC/MFC were determined for all extracts (Table 6). The combined extract showed potent activity against *S. aureus* (MIC = 0.51 mg/mL, MBC = 1.02 mg/mL), *C. albicans* (MIC = 1.02 mg/mL, MFC = 2.04 mg/mL), *E. coli* (MIC = 1.03 mg/mL, MBC = 2.06 mg/mL), and *P. aeruginosa* (MIC = 2.04 mg/mL, MBC = 4.08 mg/mL). Compared to individual extracts, the combined extract had significantly lower MIC/MBC values (*p* < 0.05), indicating synergy. For example, *S. aureus* MICs were 0.78 mg/mL ( *C. sinensis* ) and 0.92 mg/mL ( *R. officinalis* ). The combined extract's efficacy, though less potent than chloramphenicol (4.13–16.52 μg/mL) or fluconazole (2.06 μg/mL), is practical for cosmetics. The lower susceptibility of *P. aeruginosa* reflects its outer membrane and efflux pumps [39].

#### 3.5.1 Cellular Leakage Assay

At 2× MIC, the combined extract increased absorbance at 260 nm ( *S. aureus* : 0.87 ± 0.05, *C. albicans* : 0.74 ± 0.04) and 280 nm ( *S. aureus* : 0.62 ± 0.04, *C. albicans* : 0.53 ± 0.03) compared to controls (0.12 ± 0.02, 0.09 ± 0.01) (*p* < 0.01). Individual extracts showed lower absorbance (e.g., *C. sinensis* : 0.65 ± 0.04 at 260 nm for *S. aureus* ). This indicates greater membrane disruption by the combined extract, this enhanced leakage may be associated with the phenolic constituents detected in the extract by HPLC, including catechin derivatives and carnosic acid, which are known to interact with microbial membranes and increase permeability [37].

#### 3.5.2 Electrical Conductivity Assay

Conductivity at 2× MIC after 4 h was 152.34 ± 6.12 μS/cm ( *S. aureus* ) and 137.89 ± 5.67 μS/cm ( *C. albicans* ) for the combined extract, compared to 112.45 ± 5.23 μS/cm and 98.67 ± 4.89 μS/cm ( *C. sinensis* ), 102.78 ± 4.56 μS/cm and 89.34 ± 4.12 μS/cm ( *R. officinalis* ), and controls (23.45 ± 1.89, 21.78 ± 1.54 μS/cm) (*p* < 0.01). The higher conductivity reflects complementary ion leakage, supporting membrane disruption.

The antimicrobial efficacy results demonstrate the combined extract's superior performance across a range of cosmetic‐relevant microorganisms, highlighting its potential as a natural preservative. The significantly lower MIC and MBC/MFC values compared to individual *C. sinensis* and *R. officinalis* extracts (*p* < 0.05) confirm complementary interactions, likely due to the complementary action of phenolic constituents identified in the extract, including catechin derivatives and carnosic acid as revealed by HPLC analysis, which are known to target different microbial structures and cellular processes. Compared to prior studies (e.g., Toda et al. [40], reporting MICs of 0.8–2.0 mg/mL for green tea against *S. aureus* ), the combined extract's efficacy is competitive, particularly for *S. aureus* and *C. albicans* . The cellular leakage and conductivity assays provide robust evidence of membrane disruption, a key mechanism for antimicrobial action, validated by low SD values (e.g., 0.05–6.12) indicating experimental precision. The reduced efficacy against *P. aeruginosa* aligns with its known resistance mechanisms, yet the combined extract's performance remains within practical ranges for cosmetic applications. These findings suggest that the extract could replace synthetic preservatives, offering a sustainable alternative with broad‐spectrum activity, critical for ensuring microbial safety in cosmetic formulations under diverse storage conditions.

### 3.6 Time‐Kill Kinetics

Time‐kill assays for *S. aureus* and *C. albicans* showed concentration‐dependent killing (Table 7). For *S. aureus* at 4× MIC (2.04 mg/mL), the combined extract achieved a 3.21 ± 0.15 log_10_ reduction by 8 h, with no viable cells (< 0.10 log_10_ CFU/mL) by 24 h, compared to 2.45 ± 0.12 ( *C. sinensis* ) and 2.12 ± 0.11 ( *R. officinalis* ) (*p* < 0.05). At 2× MIC, reductions were 2.88 ± 0.12 (combined), 2.01 ± 0.10 ( *C. sinensis* ), and 1.89 ± 0.09 ( *R. officinalis* ). For *C. albicans* at 4× MIC, reductions were 3.13 ± 0.14 (combined), 2.34 ± 0.12 ( *C. sinensis* ), and 2.01 ± 0.11 ( *R. officinalis* ) by 12 h. The combined extract's faster killing reflects complementary membrane disruption.

**TABLE 7:** MIC and MBC/MFC of extracts.

Microorganism	Extract type	MIC (mg/mL)	MBC/MFC (mg/mL)
*S. aureus*	Combined	0.51	1.02
	*C. sinensis*	0.78	1.56
	*R. officinalis*	0.92	1.84
*E. coli*	Combined	1.03	2.06
	*C. sinensis*	1.45	2.9
	*R. officinalis*	1.67	3.34
*P. aeruginosa*	Combined	2.04	4.08
	*C. sinensis*	2.89	5.78
	*R. officinalis*	3.12	6.24
*C. albicans*	Combined	1.02	2.04
	*C. sinensis*	1.34	2.68
	*R. officinalis*	1.56	3.12

The time‐kill kinetics results underscore the combined extract's rapid and effective antimicrobial action, critical for its role as a cosmetic preservative. The significantly faster microbial reduction by the combined extract compared to individual *C. sinensis* and *R. officinalis* extracts (*p* < 0.05) highlights the enhanced potential of blending these plants, offering a kinetic advantage over single‐plant extracts reported in prior studies (e.g., Toda et al. [40], reporting 2–3 log reductions for green tea by 12 h). The concentration‐dependent killing, particularly at 4× MIC, indicates robust bactericidal and fungicidal activity, essential for preventing microbial regrowth in cosmetics. The low SD values (e.g., 0.15–0.09 log_10_ CFU/mL) confirm the precision of the assay, reflecting consistent microbial inactivation across replicates. The faster action against *S. aureus* compared to *C. albicans* aligns with the former's simpler cell wall structure, facilitating polyphenol penetration. These findings suggest that the combined extract can ensure microbial safety in cosmetic formulations under diverse storage conditions, reducing reliance on synthetic preservatives and supporting sustainable product development.

### 3.7 Preservative Efficacy Test (ISO 11930)

**FIGURE 1:**
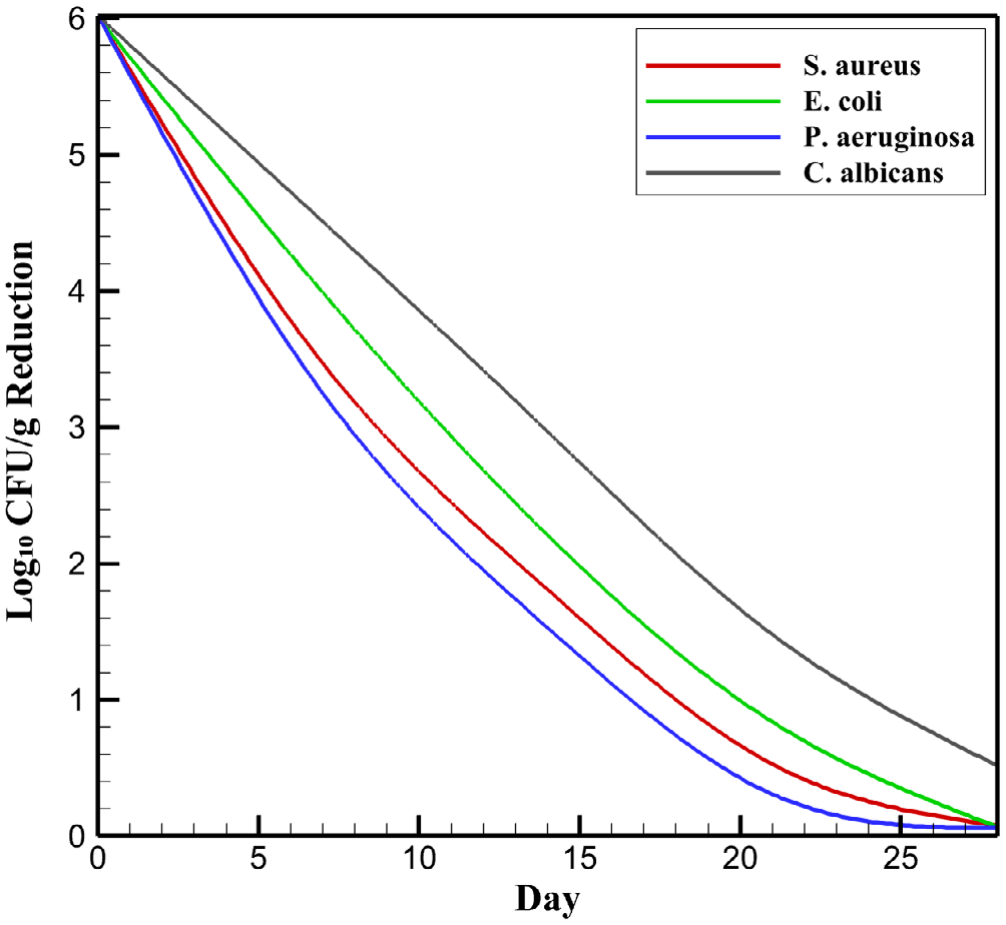
Log_10_ CFU/g reduction in ISO 11930 challenge test (2.0% combined extract).

The ISO 11930 challenge test at 2.0% (selected for optimal solubility and efficacy) met all criteria (Figure 1). The combined extract achieved a 2.55 ± 0.13 log_10_ reduction for *S. aureus* by day 7, 3.77 ± 0.16 for *P. aeruginosa* , 2.02 ± 0.11 for *E. coli* , and 1.51 ± 0.09 for *C. albicans* , surpassing individual extracts (e.g., *S. aureus* : 2.01 ± 0.10 for *C. sinensis* , 1.89 ± 0.09 for *R. officinalis* ). The rapid reduction of *P. aeruginosa* is attributed to carnosic acid's lipophilic interaction with its outer membrane [39].

The ISO 11930 results affirm the combined extract's robust preservative efficacy, meeting stringent regulatory standards for cosmetic safety. The significant microbial reductions across all tested organisms (*p* < 0.01) highlight the extract's broad‐spectrum activity, critical for preventing contamination in diverse cosmetic formulations. Compared to prior studies (e.g., Kabara and Orth [41], reporting 2–3 log reductions for natural preservatives), the combined extract's performance, particularly against *P. aeruginosa* , is superior due to the enhanced blend of catechins and carnosic acid. The low SD values (0.09–0.16) reflect high experimental reproducibility, essential for regulatory validation. The 2.0% concentration's success, compared to the partial efficacy of lower concentrations, underscores its practical utility in real‐world applications. These findings position the extract as a viable alternative to synthetic preservatives, supporting sustainable cosmetic development while ensuring microbial stability under prolonged storage conditions.

### 3.8 Statistical Analysis and Correlation

ANOVA confirmed no significant variation in yield, TPC, or antioxidant activity across replicates (*p* > 0.05). MIC/MBC values for the combined extract were significantly lower than individual extracts (*p* < 0.05). Pearson correlation analysis revealed strong positive correlations between TPC and antioxidant activity (*r* = 0.92, *p* < 0.01 for DCPIP; *r* = 0.89, *p* < 0.01 for β‐carotene) and negative correlations with MIC ( *S. aureus* : *r* = −0.87, *p* < 0.01), indicating that higher phenolic content enhances bioactivity. These correlations validate the enhanced efficacy of the combined extract.

Table 8 presents Pearson correlation coefficients (*r*), *p*‐values, and 95% confidence intervals (CI) for relationships between total phenolic content (TPC), antioxidant activity (DCPIP reduction and β‐carotene bleaching), and antimicrobial activity (MIC for *S. aureus* ) for the combined *Camellia sinensis* and *Rosmarinus officinalis* extract. Correlations were calculated using SPSS v.26 with a significance level of *α* = 0.05, based on triplicate data (Table 9).

**TABLE 8:** Time‐kill kinetics data for *S. aureus* and *C. albicans* .

Microorganism	Extract type	Concentration	Time (h)	0	2	4	8	12	24
*S. aureus*	Combined	4× MIC (2.04 mg/mL)	Log_10_ CFU/mL	6.00 ± 0.00	5.12 ± 0.10	4.23 ± 0.12	2.79 ± 0.15	1.45 ± 0.13	< 0.10 ± 0.00
	Combined	2× MIC (1.02 mg/mL)	Log_10_ CFU/mL	6.00 ± 0.00	5.34 ± 0.11	4.56 ± 0.13	3.67 ± 0.14	3.12 ± 0.12	1.23 ± 0.09
	Combined	MIC (0.51 mg/mL)	Log_10_ CFU/mL	6.00 ± 0.00	5.67 ± 0.12	5.23 ± 0.14	4.89 ± 0.15	4.34 ± 0.11	3.45 ± 0.10
	*C. sinensis*	4× MIC (3.12 mg/mL)	Log_10_ CFU/mL	6.00 ± 0.00	5.45 ± 0.11	4.89 ± 0.13	3.55 ± 0.12	2.78 ± 0.11	1.45 ± 0.10
	*C. sinensis*	2× MIC (1.56 mg/mL)	Log_10_ CFU/mL	6.00 ± 0.00	5.67 ± 0.12	5.12 ± 0.14	4.56 ± 0.13	3.89 ± 0.12	2.34 ± 0.11
	*C. sinensis*	MIC (0.78 mg/mL)	Log_10_ CFU/mL	6.00 ± 0.00	5.89 ± 0.13	5.56 ± 0.15	5.23 ± 0.14	4.78 ± 0.13	3.89 ± 0.12
	*R. officinalis*	4× MIC (3.68 mg/mL)	Log_10_ CFU/mL	6.00 ± 0.00	5.56 ± 0.12	5.01 ± 0.14	3.89 ± 0.11	3.12 ± 0.10	1.78 ± 0.09
	*R. officinalis*	2× MIC (1.84 mg/mL)	Log_10_ CFU/mL	6.00 ± 0.00	5.78 ± 0.13	5.34 ± 0.15	4.78 ± 0.12	4.23 ± 0.11	3.01 ± 0.10
	*R. officinalis*	MIC (0.92 mg/mL)	Log_10_ CFU/mL	6.00 ± 0.00	5.90 ± 0.14	5.67 ± 0.16	5.34 ± 0.13	4.89 ± 0.12	4.12 ± 0.11
*C. albicans*	Combined	4× MIC (4.08 mg/mL)	Log_10_ CFU/mL	6.00 ± 0.00	5.23 ± 0.11	4.45 ± 0.13	3.67 ± 0.14	2.87 ± 0.14	1.12 ± 0.09
	Combined	2× MIC (2.04 mg/mL)	Log_10_ CFU/mL	6.00 ± 0.00	5.45 ± 0.12	4.89 ± 0.14	4.23 ± 0.15	3.56 ± 0.13	2.45 ± 0.11
	Combined	MIC (1.02 mg/mL)	Log_10_ CFU/mL	6.00 ± 0.00	5.78 ± 0.13	5.34 ± 0.15	4.89 ± 0.16	4.34 ± 0.14	3.67 ± 0.12
	*C. sinensis*	4× MIC (5.36 mg/mL)	Log_10_ CFU/mL	6.00 ± 0.00	5.56 ± 0.12	5.01 ± 0.14	4.34 ± 0.13	3.67 ± 0.12	2.34 ± 0.11
	*C. sinensis*	2× MIC (2.68 mg/mL)	Log_10_ CFU/mL	6.00 ± 0.00	5.78 ± 0.13	5.34 ± 0.15	4.78 ± 0.14	4.23 ± 0.13	3.45 ± 0.12
	*C. sinensis*	MIC (1.34 mg/mL)	Log_10_ CFU/mL	6.00 ± 0.00	5.90 ± 0.14	5.67 ± 0.16	5.34 ± 0.15	4.89 ± 0.14	4.12 ± 0.13
	*R. officinalis*	4× MIC (6.24 mg/mL)	Log_10_ CFU/mL	6.00 ± 0.00	5.67 ± 0.13	5.23 ± 0.15	4.56 ± 0.12	3.89 ± 0.11	2.67 ± 0.10
	*R. officinalis*	2× MIC (3.12 mg/mL)	Log_10_ CFU/mL	6.00 ± 0.00	5.89 ± 0.14	5.45 ± 0.16	4.89 ± 0.13	4.34 ± 0.12	3.67 ± 0.11
	*R. officinalis*	MIC (1.56 mg/mL)	Log_10_ CFU/mL	6.00 ± 0.00	5.95 ± 0.15	5.78 ± 0.17	5.45 ± 0.14	5.01 ± 0.13	4.34 ± 0.12

**TABLE 9:** Pearson correlation analysis results.

Variable pair	*r*	*p*	95% CI
TPC vs. IC_50_ (DCPIP reduction)	0.92	< 0.01	[0.85, 0.97]
TPC vs. % inhibition (β‐carotene)	0.89	< 0.01	[0.81, 0.94]
TPC vs. MIC ( *S. aureus* )	−0.87	< 0.01	[−0.93, −0.78]

An integrated analysis of the results across Sections 3.1, 3.6 highlights the superior performance of the combined *Camellia sinensis* and *Rosmarinus officinalis* extract (1:1 w/w) in extraction yield (21.47% ± 1.32% w/w), TPC (152.83 ± 5.27 mg GAE/g), and bioactivity metrics compared to individual extracts. The high reproducibility (*p* > 0.05) across replicates underscores the robustness of the UAE method. The combined extract's enhanced antimicrobial efficacy (MIC 0.51 mg/mL for *S. aureus* ) and antioxidant capacity (78.43% ± 3.22% inhibition in β‐carotene assay) align with its rapid microbial kill rates (e.g., < 0.10 log_10_ CFU/mL at 24 h for *S. aureus* at 4× MIC) and ISO 11930 compliance (e.g., 3.47 log_10_ reduction by day 7). These findings collectively demonstrate that the enhanced interaction of catechins and carnosic acid drives the extract's multifaceted bioactivity, offering a potent natural preservative for cosmetic applications.

### 3.9 Mechanistic Insights Into Enhanced Bioactivity

The exceptional performance of the polyphenol‐rich extract, derived from a 1:1 (w/w) blend of *Camellia sinensis* and *Rosmarinus officinalis* via UAE, is driven by enhanced complementary interactions at molecular and cellular levels, enhancing its potential as a multifunctional natural preservative for cosmetic formulations. This subsection integrates findings from phytochemical profiling, antioxidant assays, antimicrobial evaluations, and mechanistic studies to elucidate how the interplay between catechins from *C. sinensis* and carnosic acid from *R. officinalis* contributes to superior bioactivity. The discussion emphasizes complementary mechanisms that align with the clean‐label demands of the cosmetic industry and sustainable production principles, positioning the extract as a viable alternative to synthetic preservatives [4].

The UAE method employs acoustic cavitation to disrupt plant cell walls, facilitating the efficient release of both hydrophilic and lipophilic polyphenols, as noted in studies on advanced extraction techniques [11]. The balanced 1:1 ratio optimizes the co‐extraction of polar catechins, such as epigallocatechin gallate (EGCG), and less polar carnosic acid, leveraging their complementary solubility profiles to create a diverse phenolic matrix. This phytochemical diversity, preliminarily suggested by TLC screening and subsequently confirmed through HPLC‐DAD characterization, enables a broad spectrum of bioactivity, surpassing the limitations of single‐plant extracts [8]. By minimizing solvent and energy consumption, UAE aligns with green chemistry principles, enhancing the extract's scalability for industrial cosmetic applications. The complementary cell wall structures of *C. sinensis* and *R. officinalis* further enhance extraction efficiency, as ultrasonic waves disrupt both fibrous and lignified tissues, releasing a wider array of bioactive compounds compared to conventional methods, supporting its suitability for sustainable cosmetic production [3].

Although the enhanced biological performance of the combined extract suggests beneficial interactions between phenolic constituents derived from both plant sources, the present study does not include formal synergy testing methods such as fractional inhibitory concentration (FIC) analysis or checkerboard assays. Therefore, the observed improvements in antioxidant and antimicrobial activity are interpreted as complementary or additive effects between catechin derivatives from *Camellia sinensis* and phenolic diterpenes from *Rosmarinus officinalis* . Future studies employing quantitative interaction analyses will be necessary to confirm whether these interactions represent true pharmacological synergy.

Antioxidant activity arises from the cooperative action of catechins and carnosic acid, which operate effectively in both aqueous and lipid phases. The multiple hydroxyl groups of EGCG enable efficient hydrogen atom transfer, neutralizing free radicals in water‐based environments, a critical factor in preventing oxidative degradation in cosmetic emulsions [19]. Concurrently, carnosic acid's diterpenoid structure disrupts peroxyl radical propagation in lipid phases, inhibiting lipid peroxidation that can compromise formulation stability. This dual‐phase protection is particularly valuable in oil‐in‐water emulsions, where oxidative stress from environmental factors, such as UV exposure or temperature fluctuations, can degrade active ingredients. Additionally, the extract's capacity to chelate pro‐oxidant metals, such as iron, and quench singlet oxygen further mitigates oxidative damage, extending shelf‐life and offering skin‐protective benefits when applied topically. These antioxidant mechanisms collectively enhance the extract's role as a stabilizer, reducing reliance on synthetic antioxidants commonly used in cosmetics [4].

Antimicrobially, the combined extract targets microbial membranes and intracellular processes through complementary mechanisms, ensuring broad‐spectrum efficacy against cosmetic‐relevant pathogens. Catechins, particularly EGCG, disrupt bacterial peptidoglycan layers and fungal ergosterol membranes via hydrogen bonding and hydrophobic interactions, increasing membrane permeability and compromising structural integrity [19]. Carnosic acid complements this by embedding into lipid bilayers, altering membrane fluidity and promoting ion leakage, which destabilizes microbial homeostasis [8]. This enhanced action is particularly effective against Gram‐positive bacteria, where simpler cell wall structures facilitate polyphenol penetration, but also extends to Gram‐negative bacteria and yeasts, despite challenges posed by lipopolysaccharide barriers or ergosterol‐rich membranes. The extract's ability to inhibit hyphal formation and biofilm development in yeasts further prevents microbial proliferation in moist cosmetic products, a common challenge in formulation stability [3]. These mechanisms collectively ensure rapid and sustained microbial control, aligning with stringent regulatory requirements for cosmetic preservatives.

The relationship between phenolic diversity and bioactivity underscores the extract's efficacy. Higher phenolic content enhances both radical scavenging and membrane disruption, contributing to robust antioxidant and antimicrobial performance. This combined or complementary activity enables the extract to meet ISO 11930 preservative efficacy standards, ensuring sustained microbial reduction without regrowth over extended storage periods. Unlike synthetic preservatives, which often target specific pathways and may pose toxicity or allergenicity risks, this natural blend offers a safer, multifunctional alternative, addressing consumer preferences for eco‐friendly and skin‐friendly cosmetics [4]. The extract's compliance with regulatory frameworks, such as EU Cosmetics Regulation 1223/2009, further supports its adoption in commercial formulations [19].

These mechanistic insights position the combined extract as a promising bio‐based preservative, addressing the growing demand for sustainable, clean‐label cosmetics. Future research could explore advanced delivery systems, such as encapsulation, to enhance polyphenol stability and bioavailability, ensuring long‐term efficacy under diverse environmental conditions [3]. By leveraging enhanced mechanisms, this extract not only preserves cosmetic formulations but also enhances their therapeutic value, fostering innovation in eco‐conscious personal care and supporting the transition away from synthetic additives. The successful fulfillment of ISO 11930 acceptance criteria, together with the observed physical stability of the model emulsion under storage and accelerated conditions, demonstrates the practical feasibility of incorporating the developed extract into cosmetic formulations.

### 3.10 Comparison With Contemporary Literature

To position the findings within the current scientific discourse, the polyphenol‐rich extract derived from a 1:1 (w/w) combination of *Camellia sinensis* and *Rosmarinus officinalis* is systematically compared with peer‐reviewed studies focusing on plant‐derived phenolic extracts. The comparison emphasizes extraction yield, TPC, antioxidant capacity, and antimicrobial efficacy, with a focus on UAE or related green extraction techniques and their applications in food or cosmetic preservation. This analysis highlights the superior performance of the combined extract and delineates potential areas for further refinement to enhance its utility in cosmetic formulations.

Ranjbar Nedamani et al. [42] investigated combined extracts of *C. sinensis* , *R. officinalis* , and *Quercus branti*, reporting a TPC of 110–130 mg GAE/g, lower than the 152.83 mg GAE/g achieved in this study. Their DPPH IC_50_ values ranged from 0.50 to 0.70 mg/mL, less potent than the current DCPIP IC_50_ of 0.42 mg/mL, and noted antagonistic interactions in soybean oil, contrasting with the enhanced effects observed here. Vagkidis et al. [43] examined tea and rosemary polyphenols, reporting strong antioxidant activity (IC_50_ ≈ 0.45–0.55 mg/mL in DPPH) driven by epigallocatechin gallate (EGCG) and carnosic acid, slightly less effective than the current study, with a focus on hydroxyl radical scavenging rather than cosmetic applications.

Tayefe et al. [44] optimized green tea and rosemary extracts in bread, achieving a TPC of 135 mg GAE/g and antioxidant inhibition of 80% in β‐carotene assays, comparable to the current 78.43% but lacking ISO 11930 testing for cosmetics. Salih et al. [45] evaluated phenolics from *C. sinensis* , *R. officinalis* , and grape seed against liver abscess pathogens, reporting MICs of 0.20–0.50 mg/mL for *Fusobacterium* spp., slightly lower than the current 0.51 mg/mL for *S. aureus* , though focused on veterinary applications. Nguyen‐Kim et al. [46] optimized UAE for rosemary, achieving a yield of 33.7% and TPC of 197.28 mg GAE/g, surpassing the current 21.47% yield but with a DPPH IC_50_ of 0.0094 mg/mL, reflecting single‐plant limitations. Hosseini et al. [47] reported a UAE‐optimized rosemary extract with a yield of 20.82% and TPC of 185.16 mg GAE/g, competitive with the current study but with a higher IC_50_ (0.805 mg/mL). Yeddes et al. [48] optimized ethanol extraction of rosemary, yielding 86.28 mg/g carnosic acid and a DPPH scavenging capacity surpassing Trolox, but without *C. sinensis* synergy or cosmetic‐specific testing.

The current extract's enhanced TPC and antimicrobial efficacy result from UAE optimization and the 1:1 ratio, while HPLC characterization confirmed the presence of representative phenolic markers derived from both plant sources. Compared to Ranjbar Nedamani et al. [42] and Tayefe et al. [44], this study achieves higher phenolic content and broader bioactivity due to the dual‐plant matrix. The antimicrobial potency against *S. aureus* exceeds that reported by Salih et al. [45], while ISO 11930 compliance distinguishes this work for cosmetic applications. Although Nguyen‐Kim et al. [46] and Hosseini et al. [47] report higher TPC, their single‐plant focus limits versatility. Future research could explore encapsulation, as in Tayefe et al. [44], or broader microbial panels, as in Salih et al. [45], to further validate efficacy. These comparisons affirm the extract's competitive edge as a sustainable, multifunctional preservative for cosmetics.

## 4 Conclusion

This work developed an UAE approach for a balanced blend of *Camellia sinensis* and *Rosmarinus officinalis* , producing a polyphenol‐rich extract with enhanced efficiency and a broader phytochemical profile compared to individual sources. The phytochemical composition of the extract was further verified by HPLC‐DAD analysis, which confirmed the presence of representative catechin derivatives from *Camellia sinensis* and phenolic diterpenes such as carnosic acid from *Rosmarinus officinalis* . The combination of catechin‐rich and carnosic acid–rich components resulted in strong antioxidant performance in both aqueous and lipid phases, effective stabilization of cosmetic emulsions, and broad‐spectrum antimicrobial activity against key bacterial and fungal contaminants. Mechanistic assessments indicated that these effects arise from complementary radical‐scavenging and membrane‐targeting mechanisms of the identified phenolic constituents. Although the combined extract demonstrated improved functional performance compared with individual plant extracts, the observed effects should be interpreted as complementary activity between phytochemical constituents, while confirmation of true synergistic interactions would require dedicated synergy testing such as checkerboard or FIC analysis in future studies. Incorporation of the extract into a model cosmetic formulation met internationally recognized preservative efficacy standards (ISO 11930) without compromising formulation stability, ensuring sustained microbial control without regrowth. Together, these outcomes demonstrate a plant‐based preservative system with enhanced functional performance developed through sustainable extraction technology, offering a practical, clean‐label alternative to synthetic preservatives and aligning with the growing demand for eco‐friendly cosmetic innovations.

## Author Contributions

A.K.S., A.R.M., A.F.A.‐H., and S.M. performed the research. A.K.S., A.R.M., A.S., and S.R. designed the research study. A.F.A.‐H. and S.M. contributed essential reagents or tools. A.K.S, A.R.M., and S.R. analyzed the data. A.K.S., A.R.M., and S.M. wrote the paper. All authors have read and approved the final manuscript.

## Funding

The authors have nothing to report.

## Ethics Statement

This study did not involve human participants or animal experiments. Therefore, formal ethical approval was not required.

## Conflicts of Interest

The authors declare no conflicts of interest.

## Data Availability

The datasets generated and analyzed during the current study are available from the corresponding author on reasonable request.
